# BindN+ for accurate prediction of DNA and RNA-binding residues from protein sequence features

**DOI:** 10.1186/1752-0509-4-S1-S3

**Published:** 2010-05-28

**Authors:** Liangjiang Wang, Caiyan Huang, Mary Qu Yang, Jack Y Yang

**Affiliations:** 1 Department of Genetics and Biochemistry, Clemson University, Clemson, SC 29634, USA; 2 J.C. Self Research Institute of Human Genetics, Greenwood Genetic Center, Greenwood, SC 29646, USA; 3 School of Electrical and Computer Engineering, Purdue University, West Lafayette, Indiana 47907 USA; 4 Center for Computational Biology and Bioinformatics, Indiana University School of Medicine, Indiana University Purdue University, Indianapolis, Indiana 46202 USA; 5 Center for Research in Biological Systems, University of California at San Diego, La Jolla, California 92093-0043, USA

## Abstract

**Background:**

Understanding how biomolecules interact is a major task of systems biology. To model protein-nucleic acid interactions, it is important to identify the DNA or RNA-binding residues in proteins. Protein sequence features, including the biochemical property of amino acids and evolutionary information in terms of position-specific scoring matrix (PSSM), have been used for DNA or RNA-binding site prediction. However, PSSM is rather designed for PSI-BLAST searches, and it may not contain all the evolutionary information for modelling DNA or RNA-binding sites in protein sequences.

**Results:**

In the present study, several new descriptors of evolutionary information have been developed and evaluated for sequence-based prediction of DNA and RNA-binding residues using support vector machines (SVMs). The new descriptors were shown to improve classifier performance. Interestingly, the best classifiers were obtained by combining the new descriptors and PSSM, suggesting that they captured different aspects of evolutionary information for DNA and RNA-binding site prediction. The SVM classifiers achieved 77.3% sensitivity and 79.3% specificity for prediction of DNA-binding residues, and 71.6% sensitivity and 78.7% specificity for RNA-binding site prediction.

**Conclusions:**

Predictions at this level of accuracy may provide useful information for modelling protein-nucleic acid interactions in systems biology studies. We have thus developed a web-based tool called BindN+ (http://bioinfo.ggc.org/bindn+/) to make the SVM classifiers accessible to the research community.

## Background

Protein-DNA and protein-RNA interactions are involved in many biological processes essential for cellular function. To understand the molecular mechanisms of the protein-nucleic acid recognition, it is important to identify the DNA or RNA-binding amino acid residues in proteins. The identification is straightforward if the structure of a protein-DNA or protein-RNA complex is known. Unfortunately, it is very expensive and time-consuming to solve the structure of a protein-DNA/RNA complex. Currently, only a few hundreds of protein-nucleic acid complexes have structural data available in the Protein Data Bank (PDB, http://www.rcsb.org/pdb/). With the rapid accumulation of sequence data, predictive methods are needed for identifying potential DNA or RNA-binding residues in protein sequences.

Several machine learning methods have been reported for predicting DNA or RNA-binding residues directly from amino acid sequences [1-3], using biochemical features of amino acid residues [4,5], and by incorporating evolutionary information in terms of position-specific scoring matrices [6-8]. Ahmad *et al*. [1] investigated representative structures of protein-DNA complexes, and used the amino acid sequences in these structures to train artificial neural networks (ANNs) for prediction of DNA-binding residues. Yan *et al*. [2] constructed Naïve Bayes classifiers for DNA-binding site prediction from amino acid identities. Naïve Bayes classifiers were also developed for predicting RNA-binding residues directly from amino acid sequences [3]. However, without using biological knowledge for classifier construction, the prediction accuracy was relatively low in these studies.

The use of evolutionary information for input encoding has been shown to improve classifier performance. Ahmad and Sarai [6] constructed ANN classifiers for DNA-binding site prediction using evolutionary information in terms of position-specific scoring matrix (PSSM). More recently, PSSM profiles have also been used to train support vector machines (SVMs) and logistic regression models for sequence-based prediction of DNA-binding residues [7,8]. For a given protein sequence, its PSSM profile can be derived from the result of a PSI-BLAST search against a large sequence database. PSSM scores indicate how well an amino acid position in the query sequence is conserved among its homologues. Since functional sites, including DNA and RNA-binding residues, tend to be conserved among homologous proteins, PSSM can provide relevant information for classifier construction. However, PSSM is rather designed for PSI-BLAST searches, and it may not contain all the evolutionary information for modelling DNA or RNA-binding sites.

In our previous studies [4,5], ANN and SVM classifiers were constructed for DNA or RNA-binding site prediction using relevant biochemical features, including the hydrophobicity index, side chain pK_a_ value, and molecular mass of an amino acid. These features were used to represent biological knowledge, which might not be learned from the training data. It was found that classifier performance was enhanced by using the biochemical features for input encoding, and the SVM classifiers outperformed the ANN predictors. Nevertheless, it is still unknown whether classifier performance can be further improved by combining the biochemical features with evolutionary information.

This study aimed to examine different descriptors of evolutionary information for DNA and RNA-binding site prediction, and to improve classifier performance by combining relevant sequence features. Three new descriptors of evolutionary information as well as PSSM were used to construct SVM classifiers, and the new descriptors were shown to improve classifier performance. Interestingly, the most accurate classifiers were obtained by combining the new descriptors with PSSM and relevant biochemical features for input encoding. The results suggest that PSSM, although useful for classifier construction, does not capture all the evolutionary information for predicting DNA and RNA-binding residues in protein sequences. A new web server called BindN+ (http://bioinfo.ggc.org/bindn+/) has been developed to make the SVM classifiers accessible to the biological research community.

## Methods

### Data preparation

Two amino acid sequence datasets, PDNA-62 and PRINR25, were derived from structural data of protein-DNA and protein-RNA complexes available at the Protein Data Bank (PDB at http://www.rcsb.org/pdb/). The PDNA-62 dataset was used to train classifiers for DNA-binding residues as in previous studies [4-7]. PDNA-62 was derived from 62 structures of representative protein-DNA complexes. The PRINR25 dataset was prepared for RNA-binding site prediction in our previous study [5]. PRINR25 was derived from 174 structures of protein-RNA complexes. Both PDNA-62 and PRINR25 had less than 25% identity among the sequences in each dataset.

As in the previous studies [1,4-6], an amino acid residue was designated as a DNA or RNA-binding site if the side chain or backbone atoms of the residue fell within a cutoff distance of 3.5 angstroms (Å) from any atoms of the DNA or RNA molecule in the complex. All the other residues were regarded as non-binding sites. Both PDNA-62 and PRINR25 were imbalanced datasets with ~15% residues labelled as binding sites and ~85% residues as non-binding sites.

### Training strategies

Support vector machines (SVMs) were trained using residue-wise data instances derived from the sequence datasets. From a sequence with *n* amino acid residues, a total of (*n* – *w* + 1) data instances were extracted, where *w* was the sliding window size. In this study, each instance consisted of eleven consecutive residues (*w* = 11) with the target residue positioned in the middle of the subsequence. An instance was labelled as 1 (positive) if the target residue was DNA/RNA-binding, or as -1 (negative) if the target residue was non-binding. The context information provided by the five neighboring residues on each side of the target residue was previously shown to be optimal for sequence-based prediction of DNA or RNA-binding residues [4,5].

To generate the input vector for training SVMs, each residue was represented with three biochemical features and several descriptors of evolutionary information (see below). The three biochemical features, including the hydrophobicity index (feature *H*), side chain pK_a_ value (feature *K*), and molecular mass (feature *M*) of an amino acid, were previously used to construct classifiers for DNA or RNA-binding site prediction [4,5].

The *SVMlight* software package available at http://svmlight.joachims.org/ was used to construct SVM classifiers. SVM, a class of relatively new machine learning algorithms, has recently been applied to a variety of biological problems for pattern classification [9]. SVM may have superior generalization power with the ability to avoid overfitting. For a given set of binary-labelled training examples, SVM maps the input space into a higher-dimensional space and seeks a hyperplane to separate the positive data instances from the negative ones [10]. The optimal hyperplane maximizes the separation margin between the two classes of training data, and is defined by a fraction of the input data instances (the so-called support vectors) close to the hyperplane. The distance measurement between the data points in the high-dimensional space is defined by the kernel function. This study used the radial basis function (RBF) kernel:

	(1)

where  and  are two data vectors, and *γ* is a training parameter. A smaller *γ* value makes the decision boundary smoother. Another parameter for SVM training is the regularization factor *C*, which controls the trade-off between low training error and large margin [10]. Different values for the *γ* and* C* parameters have been tested in this study to optimize the classifier performance.

### Extraction of evolutionary information

Considering the great complexity of protein-DNA/RNA interactions, the labelled datasets derived from the available structures are rather small in size. On the other hand, there are abundant unlabeled sequence data in public databases such as UniProt [11]. The unlabeled data contain evolutionary information about the conservation of each sequence position, and DNA/RNA-binding residues tend to be conserved among homologous proteins [12].

Position-specific scoring matrix (PSSM) has often been used as a descriptor of evolutionary information. PSSM profiles can be derived by searching a protein sequence database using the PSI-BLAST program [13]. For each position in a query sequence, there are 20 PSSM scores. The evolutionary information captured by PSSM was previously shown to improve the performance of artificial neural networks and support vector machines for DNA-binding site prediction [6,7].

**Figure 1:**
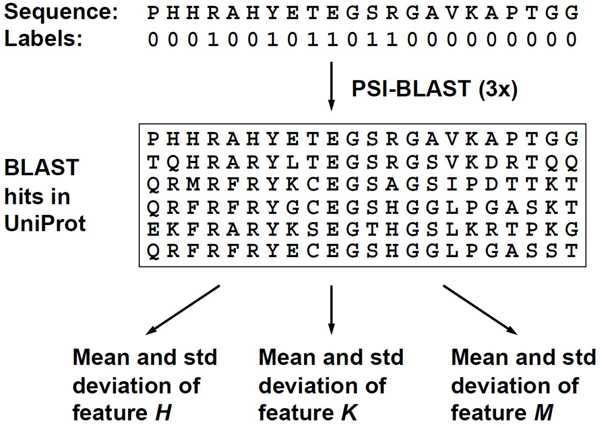
Schematic diagram for extracting evolutionary information.

However, PSSM is rather designed for general-purpose sequence comparison using PSI-BLAST, and it may not capture all the evolutionary information for predicting DNA or RNA-binding residues, which appear to have specific biochemical properties. For instance, DNA-binding residues show a distinct amino acid distribution [1-4]. Certain basic and polar amino acids are overrepresented whereas acid and hydrophobic amino acids are underrepresented in the population of DNA-binding sites. The PSSM profiles derived from PSI-BLAST search results may not precisely capture the characteristics of the amino acid distribution. Thus, new descriptors of evolutionary information have been developed in the present study to capture the conserved biochemical properties of DNA or RNA-binding residues. The approach is illustrated in Figure 1.

For a given protein sequence *p*, its homologues *B_p_* = {*b_1_*, *b_2_*, …, *b_j_*) in a reference database can be retrieved and aligned to *p* using PSI-BLAST. In this study, the protein sequences in UniProtKB (http://www.pir.uniprot.org/) were used as the reference database, and PSI-BLAST was run for three iterations with the E-value threshold set to 1e-5. The sequence alignment was then used to compute the mean  and standard deviation *σ* of a feature *X* for each residue *a_i_* in the protein sequence *p*:

	(2)

	(3)

where  is the value of feature *X* for the amino acid residue in *b_j_*, which is aligned to *a_i_* at position *i* in the query sequence *p*.

Although *X* can be any biological feature with a numerical domain, the three biochemical features relevant for DNA and RNA-binding site prediction have been investigated in this study, that is, . The new descriptors of evolutionary information can be defined as follows:

(1) : The mean and standard deviation of the *H* feature values for each residue *a_i_* in the sequence *p*. Hydrophobicity (*H*) is a key factor in amino acid side chain packing and protein folding. Hydrophobic amino acids, which are often located inside proteins, are underrepresented at the DNA interaction interfaces [1-4]. Thus, if a residue has the greater mean of hydrophobicity with less standard deviation in the sequence alignment, the residue in the query sequence is less likely to be located at the interaction interface.

(2) : measures how well the side chain pK_a_ value (*K*) of an amino acid residue is conserved among the homologous sequences in the alignment. The side chain pK_a_ determines the ionization state of a residue. Since the phosphate groups of nucleic acids are negatively charged, the ionization state of amino acid side chains affects the interaction with DNA or RNA molecules. Amino acid residues with positively charged side chains (*e.g.*, arginine) are overrepresented at the interaction interface. In other words, if a residue has the greater mean of feature *K* with less standard deviation in the sequence alignment, the residue in the query sequence is more likely to be a DNA or RNA-binding residue.

(3) : Each amino acid has a unique value of molecular mass (feature *M*), which is closely related to the volume of space occupied by the residue in protein structures. DNA or RNA-binding residues may have the size constraint to be fitted into the interaction interface, and the mean and standard deviation of *M* may be used to represent the evolutionary information for the size constraint.

### Classifier evaluation

A fivefold cross-validation approach was used to evaluate the performance of SVM classifiers. Positive and negative instances were distributed randomly into five folds. In each of the five iterative steps, four of the five folds were used to train a classifier, and then the classifier was evaluated using the holdout fold (test data). The predictions made for the test instances in all the five iterations were combined and used to compute the following performance measures:

	(4)

	(5)

	(6)

	(7)

where *TP* is the number of true positives; *TN* is the number of true negatives; *FP* is the number of false positives; and *FN* is the number of false negatives. Since the datasets used in this study are imbalanced, both sensitivity and specificity are also computed from prediction results. Furthermore, the average of sensitivity and specificity, referred to as strength in this paper, has been shown to provide a fair measure of classifier performance [1-4].

Matthews Correlation Coefficient (MCC) is commonly used as a measure of the quality of binary classifications [14]. It measures the correlation between predictions and the actual class labels. However, for imbalanced datasets, different tradeoffs of sensitivity and specificity may give rise to different MCC values for the same classifier. MCC is defined as:

	(8)

The Receiver Operating Characteristic (ROC) curve is probably the most robust approach for classifier evaluation and comparison [15]. The ROC curve is drawn by plotting the true positive rate (*i.e.*, sensitivity) against the false positive rate, which equals to (1 – specificity). In this work, the ROC curve has been generated by varying the output threshold of a classifier and plotting the true positive rate against false positive rate for each threshold value. The area under the ROC curve (AUC) can be used as a reliable measure of classifier performance [16]. Since the ROC plot is a unit square, the maximum value of AUC is 1, which is achieved by a perfect classifier. Weak classifiers have AUC values close to 0.5.

## Results and discussion

### DNA-binding site prediction

The three biochemical features, including the hydrophobicity index (feature *H*), side chain pK_a_ value (*K*), and molecular mass (*M*) of an amino acid, were previously used to construct SVM classifiers for DNA or RNA-binding residues [5], and these classifiers have been used by the BindN web server (available at http://bioinfo.ggc.org/bindn/). Similar performance measures were also obtained in this study. As shown in Table 1, the SVM classifier without using any evolutionary information achieved 70.0% prediction strength with 69.5% sensitivity and 70.6% specificity. The Matthews correlation coefficient (MCC) of this classifier was 0.295, and ROC AUC = 0.761. Different SVM training parameters were tested, and the optimal parameter settings were based on the highest prediction strength and ROC AUC. It should be noted that the dataset was imbalanced, and the overall accuracy could be misleading (*e.g.*, ~85% accuracy by simply predicting all the residues as negatives).

**Table 1:** Effect of evolutionary information on DNA-binding site prediction.

Evolutionary Information	Accuracy(%)	Sensitivity(%)	Specificity(%)	Strength(%)	MCC	ROC AUC
None	70.4	69.5	70.6	70.0	0.295	0.761
	72.0	71.3	72.1	71.7	0.323	0.779
	74.8	73.4	75.0	74.2	0.365	0.813
	71.1	70.0	71.3	70.7	0.306	0.771
	76.2	72.4	76.8	74.6	0.377	0.817
PSSM	77.7	74.8	78.2	76.5	0.409	0.849
PSSM +	79.0	77.3	79.3	78.3	0.440	0.859

Classifier performance was improved to varying levels when each of the three new descriptors of evolutionary information was added to the biochemical features for input encoding. As shown in Table 1, the  descriptor (the mean and standard deviation of feature *K*) gave rise to the highest performance with 74.2% prediction strength (73.4% sensitivity and 75.0% specificity), MCC = 0.365 and ROC AUC = 0.813. The classifier using all the three new descriptors (, and ) achieved slightly better performance with 74.6% prediction strength (72.4% sensitivity and 76.8% specificity), MCC = 0.377 and ROC AUC = 0.817. Therefore, the use of the three new evolutionary information descriptors for input encoding was found to improve classifier performance.

Position-specific scoring matrix (PSSM) was previously shown to improve the accuracy of DNA-binding site prediction [6-8]. In this study, the SVM classifier constructed using PSSM in addition to the biochemical features achieved high performance with 76.5% prediction strength (74.8% sensitivity and 78.2% specificity), MCC = 0.409 and ROC AUC = 0.849. Interestingly, the most accurate classifier was obtained by combining PSSM with the new descriptors of evolutionary information for input encoding. This classifier achieved 78.3% prediction strength (77.3% sensitivity and 79.3% specificity), MCC = 0.440 and ROC AUC = 0.859 (Table 1).

The results suggest that although PSSM can be used to improve classifier performance, it does not capture all the evolutionary information for DNA-binding site prediction. While PSSM scores indicate whether an amino acid residue is conserved among homologous sequences, the three new descriptors can be used to represent the conservation of the relevant biochemical properties for DNA-binding residues. However, since classifier performance is improved only slightly by combining PSSM with the new descriptors, it is likely that the evolutionary information captured by the different descriptors may be partially overlapping.

**Figure 2:**
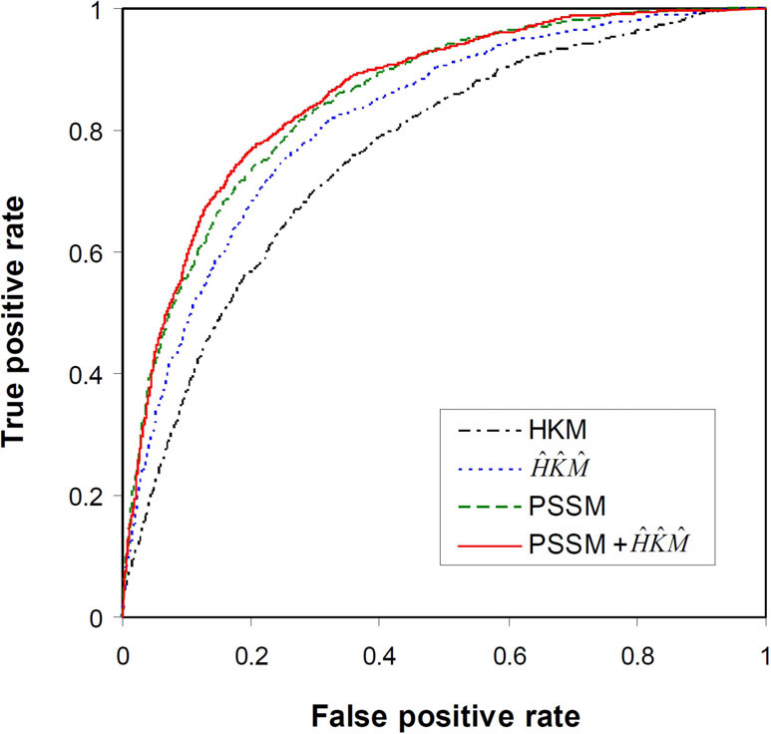
ROC analysis to show the effect of evolutionary information on prediction of DNA-binding residues.

The ROC curves of four SVM classifiers are shown in Figure 2. In general, the ROC curve of a more accurate classifier is closer to the left-hand and top borders of the plot. Thus, the three classifiers using evolutionary information are clearly better than the SVM classifier constructed with only the biochemical features (*HKM*). The classifier using PSSM is slightly better than the classifier constructed with the new descriptors (), and the most accurate classifier appears to the SVM using all the different descriptors of evolutionary information (PSSM +).

### RNA-binding site prediction

The biochemical properties of RNA-binding residues are similar but not identical to those of DNA-binding residues [17,18]. It is thus interesting to investigate how RNA-binding site prediction is affected by using the different descriptors of evolutionary information. The SVM classifier constructed with only the biochemical features achieved 68.0% prediction strength (66.0% sensitivity and 69.9% specificity), MCC = 0.265 and ROC AUC = 0.741 (Table 2). This classifier has been used by the BindN web server for RNA-binding site prediction.

**Table 2:** Effect of evolutionary information on RNA-binding site prediction.

Evolutionary Information	Accuracy(%)	Sensitivity(%)	Specificity(%)	Strength(%)	MCC	ROC AUC
None	69.3	66.0	69.9	68.0	0.265	0.741
	70.1	67.4	70.5	69.0	0.281	0.757
	73.4	66.5	74.6	70.5	0.312	0.774
	69.2	66.6	69.6	68.1	0.267	0.744
	74.6	67.4	75.8	71.6	0.331	0.784
PSSM	76.8	71.5	77.7	74.6	0.380	0.818
PSSM +	77.7	71.6	78.7	75.2	0.393	0.825

Classifier performance was improved by using each of the new descriptors of evolutionary information. In particular, the use of descriptor  resulted in slightly better performance with 70.5% prediction strength (66.5% sensitivity and 74.6% specificity), MCC = 0.312 and ROC AUC = 0.774. The performance was improved to 71.6% prediction strength (67.4% sensitivity and 75.8% specificity), MCC = 0.331 and ROC AUC = 0.784 when all the three new descriptors of evolutionary information were used for classifier construction (Table 2).

The use of PSSM was also found to significantly improve RNA-binding site prediction, and the classifier achieved 74.6% prediction strength (71.5% sensitivity and 77.7% specificity), MCC = 0.380 and ROC AUC = 0.818. Nevertheless, the classifier constructed using all the descriptors of evolutionary information (PSSM, , and ) appeared to give the best predictive performance with 75.2% prediction strength (71.6% sensitivity and 78.7% specificity), MCC = 0.393 and ROC AUC = 0.825 (Table 2).

**Figure 3:**
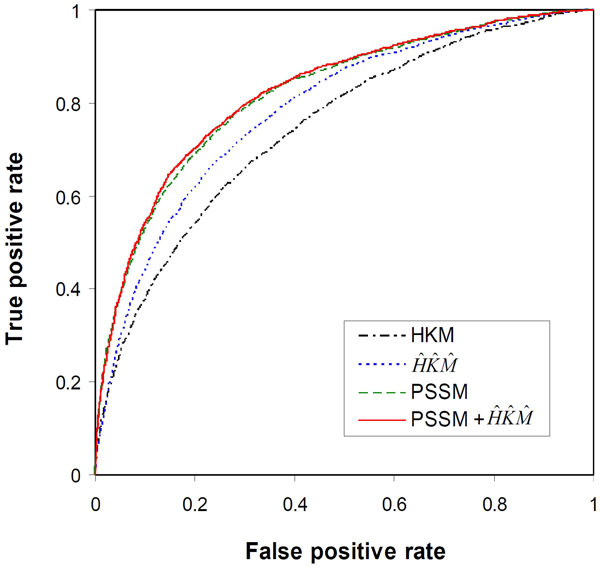
ROC analysis to show the effect of evolutionary information on prediction of RNA-binding residues.

The results have been further confirmed by the ROC analysis. As shown in Figure 3, the SVM classifier with PSSM + is slightly better than the classifier with PSSM, and all the three classifiers using evolutionary information are clearly better than the SVM trained with only the biochemical features (*HKM*). Therefore, the various descriptors of evolutionary information appear to have similar effects on both DNA and RNA-binding site prediction.

### Comparison with previous classifiers

The best SVM classifiers developed in this study are compared favourably with the other existing predictors. For DNA-binding site prediction, DBS-PSSM [6], a PSSM-based artificial neural network predictor constructed using the PDNA-62 dataset, was shown to give 68.2% sensitivity and 66.0% specificity. By contrast, the best classifier in this study achieved 77.3% sensitivity and 79.3% specificity (Table 1).

The DP-Bind system provided several classifiers for DNA-binding site prediction, and these classifiers were also constructed using the PDNA-62 dataset. The PSSM-based SVM classifier of DP-Bind achieved 76.9% sensitivity and 74.7% specificity with ROC AUC = 0.836 on imbalanced test datasets [7]. The best performance was achieved by the PSSM-based kernel logistic repression predictor [8], and the average of sensitivity and specificity reached 76.5%. In this study, the best SVM classifier achieved 78.3% prediction strength and ROC AUC = 0.859 (Table 1).

Yan *et al.*[2] developed a Naïve Bayes classifier for DNA-binding residues, and evolutionary information was not used for input encoding. The Matthews correlation coefficient of the Naïve Bayes classifier reached 0.28, which is significantly lower than that of the present study (MCC = 0.440, Table 1).

For RNA-binding site prediction, Terribilini *et al.*[3] reported a Naïve Bayes classifier that could predict at 38% sensitivity and 93% specificity (65.5% prediction strength). The highest MCC value of the Naïve Bayes classifier was 0.35. In contrast, this study achieved 75.2% prediction strength and MCC = 0.393 (Table 2). With the specificity level set to 93.0% on the ROC curve (Figure 3), the best SVM classifier had 47.0% sensitivity and MCC = 0.421. Thus, the SVM classifier developed in this study appears to be more accurate than the Naïve Bayes model [3] for RNA-binding site prediction.

### Web server description

To make the SVM classifiers accessible to the biological research community, we have developed the BindN+ web server (http://bioinfo.ggc.org/bindn+/). The web interface of BindN+ is similar to that of our previous system, BindN [5]. Users can enter an amino acid sequence in FASTA format; choose the type of prediction to be made for either DNA or RNA-binding residues; and specify the desired level of sensitivity or specificity for the prediction result. The system performs a three-iteration PSI-BLAST search against the UniProtKB database to extract evolutionary information as described in Methods. The query sequence is encoded using the three biochemical features (*H*, *K* and *M*), PSSM, and the new descriptors of evolutionary information (, and ). The most accurate SVM classifier constructed in this study is then used to scan the query sequence for putative DNA or RNA-binding residues. To make predictions, the user-defined level of sensitivity or specificity is used to choose the output threshold of the SVM model according to its ROC curve shown in Figure 2 or Figure 3.

**Figure 4:**
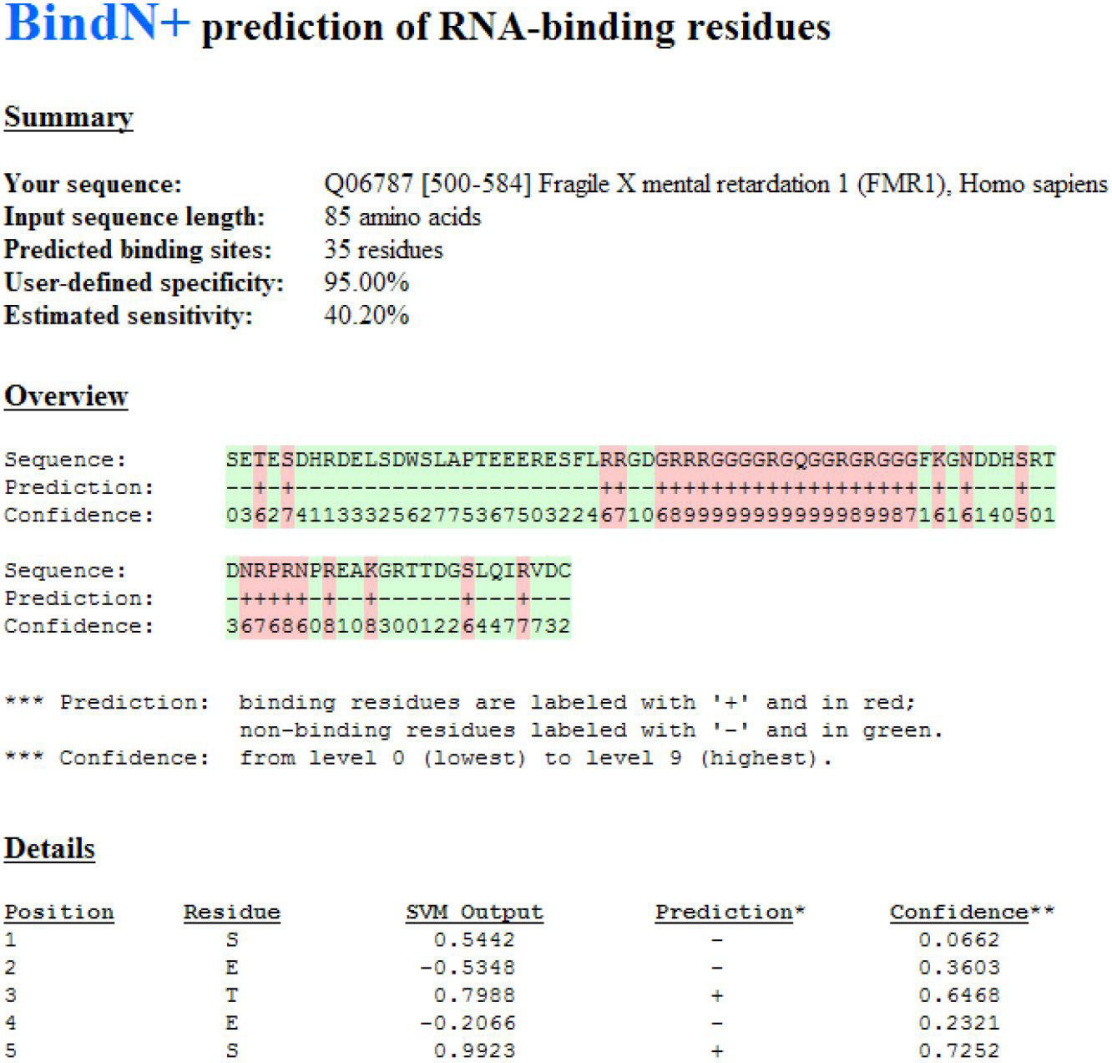
Sample output from the BindN+ web server.

The output report of BindN+ includes a summary of the prediction result, an overview of the predicted DNA or RNA-binding residues, and detailed information about the prediction for each residue. A sample report is shown in Figure 4 for the RGG box and flanking sequence of the human fragile X mental retardation 1 (FMR1) protein. Mutations in FMR1 cause the most common form of inherited mental retardation, and the RGG box has been shown to bind G-quartet mRNAs important for neuronal function [19]. For the summary, the estimated sensitivity (or specificity) is computed using the classifier’s ROC curve. In the example, the user-defined specificity was 95.00%, and the estimated sensitivity was 40.20% (Figure 4). The overview can be used to examine the distribution of putative binding residues along the query sequence. Positive predictions (putative binding residues) are labelled with ‘+’ and highlighted in red, whereas negative predictions are labelled with ‘-’ in green. In the example, the RGG box (RGGGGRGQGGRGRGG) and some neighbouring residues are predicted to interact with RNA. The confidence of prediction is computed as follows. Let *o* be the output of the SVM classifier, *sn* and *sp* be the corresponding sensitivity and specificity, respectively, on the classifier’s ROC curve, and *t* be the output threshold. Then, for a positive prediction (*o* ≥ *t*), its confidence value is set to (1 – *sn*). For a negative prediction (*o* <*t*), its confidence value is set to (1 – *sp*). The confidence value indicates where the SVM output is ranked when compared with all the true positive or true negative predictions in the cross-validation. For example, the fifth residue (S) of the input sequence in Figure 4 gives the SVM output equal to 0.9923 and has the confidence for positive prediction equal to 0.7252, which indicates that 72.52% of the RNA-binding residues in the training dataset (PRINR25) have SVM outputs less than 0.9923. For the prediction overview, the confidence level is computed as the floor of (10 × confidence) so that it ranges from the lowest level 0 to the highest level 9 for the purpose of presentation.

BindN+ represents a significant upgrade to the previous web server BindN, which was based on SVM models constructed with the relevant biochemical features [5]. BindN has been frequently accessed, and the prediction results have been shown to provide useful information for biological research [20]. Since our approach does not require structural information for binding site prediction, BindN+ can be used for genome-wide analyses of DNA and RNA-binding proteins. The analytical results may provide useful information for systematic understanding of protein-nucleic acid interactions.

## Conclusions

In this study, several descriptors of evolutionary information have been examined for sequence-based prediction of DNA and RNA-binding residues. The new descriptors of evolutionary information have been shown to improve classifier performance. Interestingly, the most accurate classifiers have been obtained by combining the new descriptors, PSSM and relevant biochemical features for input encoding. The results suggest that although PSSM can be used to improve classifier performance, it does not capture all the evolutionary information for DNA and RNA-binding site prediction. The SVM classifiers developed in this study are compared favourably with the other existing predictors. Thus, a new web server called BindN+ (http://bioinfo.ggc.org/bindn+/) has been developed to make the SVM classifiers publicly available. We anticipate that BindN+ can provide a useful tool for modelling protein-nucleic acid interactions in systems biology studies.

## Competing interests

The authors declare that they have no competing interests.

## Authors’ contributions

LW initiated and designed the study. LW and CH conducted the data analysis. LW drafted the manuscript. MQY and JYY provided valuable insights on biomolecular interactions and systems biology modeling, participated in result interpretation and manuscript preparation. All authors have reviewed the final version and agreed on the content. 
